# Supplementary material for: Identifying and Avoiding Risk of Bias in Caries Diagnostic Studies
Source: J Clin Med. 2021 Jul 22;10(15):3223. doi: 10.3390/jcm10153223 (PMC8347423; doi:10.3390/jcm10153223)
Supplement: Supplementary file 1 [file jcm-10-03223-s001.zip › jcm-1250982-supplementary.pdf]

Journal of
Clinical Medicine

Supplementary material

Criteria for risk of bias (RoB) assessment in caries diagnostic studies

Signalling questions	RoB	Description of the criteria (Domain 1)				
Patient selection bias<br>1. Is an eligible sample selected from the study participants/population?<br><br>*In vivo studies only*	Indicators for low RoB	The eligible sample from the study participants/population is representative of the patients for whom the results of the study was applicable. The eligible sample is homogenous, enrolled consecutively or randomly, and obtained from the research question, e.g., PIRD.				
	Indicators for high RoB	The eligible sample is not representative, the clinical indication for the application of the diagnostic test(s) is not completely described, and the sample is not enrolled consecutively or randomly; there is no research question.				
	Response options	Yes<br>(low RoB)	Most likely yes<br>(low RoB)	Most likely no<br>(high RoB)	No<br>(high RoB)	No information<br>(unclear)
Tooth selection bias<br>2. Is an eligible sample of teeth selected?	Indicators for low RoB	Eligible selection of the target teeth and surfaces:<br>- In vitro/in vivo studies on *occlusal* caries detection = permanent molars/primary molars<br>- In vitro/in vivo studies on *proximal* caries detection = permanent molars & premolars/primary molars.				
	Indicators for high RoB	The selected teeth and surfaces are not homogeneous; there is over/underrepresentation of at least one group of teeth, a mixture of posterior and anterior teeth, a mixture of permanent and primary teeth or a mixture of occlusal surfaces from premolars and molars.				
	Response options	Yes<br>(low RoB)	Most likely yes<br>(low RoB)	Most likely no<br>(high RoB)	No<br>(high RoB)	No information<br>(unclear)

Signalling questions	RoB	Description of the criteria (Domain 1)				
Spectrum bias<br>3. Is an appropriate spectrum of caries lesions selected?	Indicators for low RoB	All stages of caries (e.g., sound/enamel/dentin caries/caries at least in the inner half of the dentin or non-cavitated/cavitated caries) are included. The sampled caries spectrum should be pre-assessed.				
	Indicators for high RoB	At least one stage of caries is excluded or under/over-represented in the study.				
	Response options	Yes (low RoB)	Probably yes (low RoB)	Probably no (high RoB)	No (high RoB)	No information (unclear)
Sample size<br>4. Is the sample size appropriate for validity and reproducibility testing?	Indicators for low RoB	The sample size is statistically determined.				
	Indicators for high RoB	There is no sample size calculation, etc.				
	Response options	Yes (low RoB)	Probably yes (low RoB)	Probably no (high RoB)	No (high RoB)	No information (unclear)

<table>
<tr><th>Signalling questions</th><th>RoB</th><th colspan="5">Description of the criteria (Domain 2)</th></tr>
<tr><td rowspan="3">Index test criteria
5. Do/does the index test(s) correctly classify the target condition?</td><td>Indicators for low RoB</td><td colspan="5">Exact pre-definition/prescription of the criteria used, thresholds for the index test. Correct usage of the index test(s) according to latest recommendations (justified on the basis of the references).</td></tr>
<tr><td>Indicators for high RoB</td><td colspan="5">Modifications of the index test(s), mis-usage, misinterpretation.</td></tr>
<tr><td>Response options</td><td>Yes
(low RoB)</td><td>Probably yes
(low RoB)</td><td>Probably no
(high RoB)</td><td>No
(high RoB)</td><td>No information
(unclear)</td></tr>
<tr><td rowspan="3">Blinding bias
(index test)
6. Are the index test(s) data interpreted without knowledge of the results of the reference standard?</td><td>Indicators for low RoB</td><td colspan="5">Appropriate blinding of the examiners who are making the decisions/diagnoses from index test(s), e.g., at least a one-week interval between examinations, randomized/shuffled allocation of the order of specimen/images and/or inclusion of multiple examiners who are performing only one test each.</td></tr>
<tr><td>Indicators for high RoB</td><td colspan="5">Insufficient blinding. Same examiner performed multiple tests.</td></tr>
<tr><td>Response options</td><td>Yes
(low RoB)</td><td>Probably yes
(low RoB)</td><td>Probably no
(high RoB)</td><td>No
(high RoB)</td><td>No information
(unclear)</td></tr>
<tr><td rowspan="3">Calibration bias
(index test)
7. Were the examiners trained/calibrated for the performing the index test(s)?</td><td>Indicators for low RoB</td><td colspan="5">Details and outcomes of the calibration training, including the Kappa values for intra- and inter-examiner reliability, are given. Calibration training must include an independent sample of individuals or teeth. Calibration data should not be interpreted/misunderstood as intra-examiner reliability.</td></tr>
<tr><td>Indicators for high RoB</td><td colspan="5">Insufficient training/calibration.</td></tr>
<tr><td>Response options</td><td>Yes
(low RoB)</td><td>Probably yes
(low RoB)</td><td>Probably no
(high RoB)</td><td>No
(high RoB)</td><td>No information
(unclear)</td></tr>
</table>

<table>
<tr><th>Signalling questions</th><th>RoB</th><th colspan="5">Description of the criteria (Domain 3)</th></tr>
<tr><td rowspan="3">Reference test criteria
8. Does the reference test correctly classify the target condition?</td><td>Indicators for low RoB</td><td colspan="5">Usage of an optimal (“perfect”) reference standard, e.g., histology, microradiography or μCT. Exact pre-definition/prescription of the used criteria, thresholds for the reference test. Correct usage of the reference test according to the latest recommendations (justified on the basis of references). The reference test is conditionally independent of the index tests.</td></tr>
<tr><td>Indicators for high RoB</td><td colspan="5">Usage of a sub-optimal (“imperfect”) reference standard, e.g., radiography. Modifications of the reference test; mis-usage; misinterpretation. The reference test is conditionally not independent of the index test. Differential misclassification – the error rate is associated with the index test results. Non-differential misclassification – the error rate is independent of the index test results, but this can underestimate SE and SP.</td></tr>
<tr><td>Response options</td><td>Yes
(low RoB)</td><td>Probably yes
(low RoB)</td><td>Probably no
(high RoB)</td><td>No
(high RoB)</td><td>No information
(unclear)</td></tr>
<tr><td rowspan="3">Blinding bias
(reference test)
9. Is the reference test data interpreted without knowledge of the results of the index test(s)?</td><td>Indicators for low RoB</td><td colspan="5">Appropriate blinding of the examiners who are making the decisions/diagnoses from reference test(s). For example, there is at least a one-week interval between examinations, randomized/shuffled allocation of the order of specimen/images and/or inclusion of multiple examiners, who are performing only one test each, with unawareness of the outcome of index test(s).</td></tr>
<tr><td>Indicators for high RoB</td><td colspan="5">Insufficient blinding. For example, the same examiner performs multiple tests within a few days.</td></tr>
<tr><td>Response options</td><td>Yes
(low RoB)</td><td>Probably yes
(low RoB)</td><td>Probably no
(high RoB)</td><td>No
(high RoB)</td><td>No information
(unclear)</td></tr>
<tr><td rowspan="2">Calibration bias
(reference test)</td><td>Indicators for low RoB</td><td colspan="5">Details and outcomes of the calibration training, including Kappa values for intra- and inter-examiner reliability, are given. Calibration training must include an independent sample of individuals or teeth. Calibration data should not be interpreted/misunderstood as intra-examiner reliability.</td></tr>
<tr><td>Indicators for high RoB</td><td colspan="5">Insufficient training/calibration.</td></tr>
</table>

10. Are the examiners trained/calibrated for performing the reference test(s)?	Response options	Yes (low RoB)	Probably yes (low RoB)	Probably no (high RoB)	No (high RoB)	No information (unclear)
**Signalling questions**	**RoB**	**Description of the criteria (Domain 4)**				
Incorporation bias 11. Are the reference test(s) performed separately from the index test(s)?	Indicators for low RoB	The reference and index test are performed separately.				
	Indicators for high RoB	The index test is incorporated in a (composite) reference test; the result of the index test is explicitly used as a criterion for the reference test.				
	Response options	Yes (low RoB)	Probably yes (low RoB)	Probably no (high RoB)	No (high RoB)	No information (unclear)
Partial verification bias 12. Do all patients/teeth/surfaces undergo both the reference tests and the index tests?	Indicators for low RoB	Ensure that all patients/teeth/surfaces undergo both the reference tests and the index tests.				
	Indicators for high RoB	Identified when a non-random set of patients/teeth/surfaces does not undergo the reference test and the verification rate depends on the index test results.				
	Categories of RoB	Yes (low RoB)	Probably yes (low RoB)	Probably no (high RoB)	No (high RoB)	No information (unclear)
Differential verification bias 13. Do all patients/teeth/surfaces receive the same reference standard?	Indicators for low RoB	Ensure that all patients/teeth/surfaces receive the same reference standard.				
	Indicators for high RoB	Identified when a non-random set of patients/teeth/surfaces is verified with a second or third reference test, especially when this selection depends on the index test result.				

Response options	Yes (low RoB)	Probably yes (low RoB)	Probably no (high RoB)	No (high RoB)	No information (unclear)

Signalling questions	RoB	Description of the criteria (Domain 4)				
Bias in the analysis 14. Are all patient/teeth/surfaces, uninterpretable or intermediate test results and withdrawals included in the analysis?	Indicators for low RoB	All patients (teeth) who entered the study are accounted for, and all uninterpretable or intermediate test results and withdrawals (including lost specimens of the teeth) are explained.				
	Indicators for high RoB	Not all patients (teeth) who entered the study are accounted for, and not all uninterpretable or intermediate test results and withdrawals (including lost specimens of the teeth) are explained.				
	Response options	Yes (low RoB)	Probably yes (low RoB)	Probably no (high RoB)	No (high RoB)	No information (unclear)
Validity bias 15. Are the validation of results for the test method(s) included in the analysis?	Indicators for low RoB	Full presentation of results: Cross-tabulation (or distribution) of the index and reference test results by the reference standard results. Estimates of diagnostic accuracy and their precision are included (SE, SP, Az value).				
	Indicators for high RoB	Insufficient/incomplete information's, e.g., missing 2x2 contingency tables and/or SE, SP, Az values. Incorrect statistics. Biased interpretation.				
	Response options*	Yes (low RoB)	Probably yes (low RoB)	Probably no (high RoB)	No (high RoB)	No information (unclear)
Reproducibility bias 16. Are the reliability data of results for the test method(s) included in analysis?	Indicators for low RoB	Full presentation of results: Intra- and inter-examiner reliability for all examiners and for all teeth. Correct statistical procedures, e.g., Kappa values, Bland-Altman-Plots, etc. Data from the calibration training have not been mis/interpreted as reliability.				
	Indicators for high RoB	Insufficient/incomplete information on intra- and inter-examiner reliability or incorrect statistics. Data for calibration purposes only. Biased interpretation.				
	Response options	Yes (low RoB)	Probably yes (low RoB)	Probably no (high RoB)	No (high RoB)	No information (unclear)
